# Inhibiting the Evolution of Antibiotic Resistance

**DOI:** 10.1016/j.molcel.2018.10.015

**Published:** 2019-01-03

**Authors:** Mark N. Ragheb, Maureen K. Thomason, Chris Hsu, Patrick Nugent, John Gage, Ariana N. Samadpour, Ankunda Kariisa, Christopher N. Merrikh, Samuel I. Miller, David R. Sherman, Houra Merrikh

**Affiliations:** 1 Department of Microbiology, University of Washington, Seattle, WA, USA; 2 Molecular and Cellular Biology Graduate Program and Medical Scientist Training Program, University of Washington, Seattle, WA, USA; 3 Department of Genome Sciences, University of Washington, Seattle, WA, USA; 4 Center for Infectious Disease Research, Seattle, WA, USA; 5 Interdiscipinary Program of Pathobiology, Department of Global Health, University of Washington, Seattle, WA, USA

**Keywords:** antimicrobial resistance, antibiotic resistance, transcription-coupled repair, Mfd, evolution, Mycobacteria, hypermutator, anti-evolution

## Abstract

Efforts to battle antimicrobial resistance (AMR) are generally focused on developing novel antibiotics. However, history shows that resistance arises regardless of the nature or potency of new drugs. Here, we propose and provide evidence for an alternate strategy to resolve this problem: inhibiting evolution. We determined that the DNA translocase Mfd is an “evolvability factor” that promotes mutagenesis and is required for rapid resistance development to all antibiotics tested across highly divergent bacterial species. Importantly, hypermutator alleles that accelerate AMR development did not arise without Mfd, at least during evolution of trimethoprim resistance. We also show that Mfd’s role in AMR development depends on its interactions with the RNA polymerase subunit RpoB and the nucleotide excision repair protein UvrA. Our findings suggest that AMR development can be inhibited through inactivation of evolvability factors (potentially with “anti-evolution” drugs)—in particular, Mfd—providing an unexplored route toward battling the AMR crisis.

## Highlights

• The bacterial transcription-coupled repair (TCR) factor Mfd promotes mutagenesis
• Mfd-driven mutagenesis accelerates the evolution of antimicrobial resistance (AMR)
• The rapid evolution of AMR requires Mfd’s interaction with RpoB and UvrA
• Mfd may be an ideal target for “anti-evolution” drugs that inhibit AMR development

## Graphical Abstract

**Figure:**
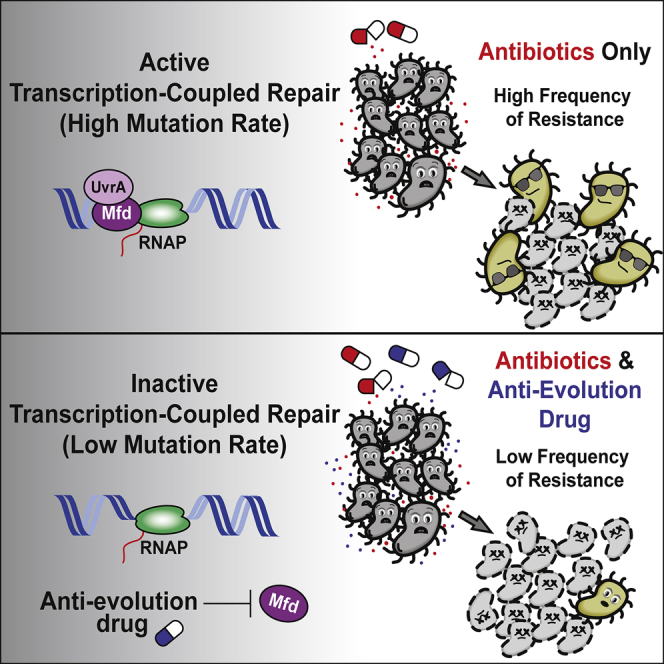

## Teaser

Bacterial evolution drives antimicrobial resistance (AMR) development. We identify the protein Mfd as a highly conserved “evolvability factor” that increases mutagenesis and the capacity of bacteria to evolve antibiotic resistance. We propose inhibiting the activity of evolvability factors through “anti-evolution” drugs during antibiotic treatment to ameliorate the global AMR crisis.

## Introduction

The battle between antimicrobial-resistant pathogens and antibiotic therapy is an evolutionary arms race—one that we are currently losing. Consequently, antimicrobial resistance (AMR)-related deaths have reached alarming rates throughout the world. Estimates suggest that at least 700,000 people die annually from drug-resistance infections; this number could rise to 10 million by 2050, far surpassing cancer as the major cause of death worldwide (O’Neill, 2014). Most efforts to resolve AMR are geared toward the development of novel antibiotics, yet resistance has arisen to every antibiotic used in the clinic. Innovative strategies to reduce the rise of drug-resistant pathogens are therefore a necessary public health concern.

For many pathogens and antibiotic classes, *de novo* mutations play a critical role in AMR development. For example, in the case of *Mycobacterium tuberculosis* (*Mtb*), the causative agent of tuberculosis (TB), AMR acquisition arises exclusively through chromosomal mutations (Almeida Da Silva and Palomino, 2011). Given the alarming global burden of TB drug resistance in addition to the rise of chromosomally acquired AMR in many other pathogens, reducing the mutational capacity of organisms could significantly inhibit their ability to develop AMR. This approach requires the identification and subsequent inhibition of active factors that increase mutation rates. We term these proteins “evolvability factors” given that they can promote evolution by increasing mutation rates (either directly or indirectly).

The DNA translocase protein Mfd is highly conserved across bacterial phyla, suggesting that it plays an important physiological role in cells. Like its functional analog CSB in humans, Mfd’s main function has long been thought to be in the initiation of nucleotide excision repair (NER—which repairs bulky lesions on DNA) at sites of stalled RNA polymerases (RNAP) (Hanawalt and Spivak, 2008). This mechanism is referred to as transcription-coupled repair (TCR). Comprehensive biochemical studies have provided insight into the various functions of Mfd, including its role in the recruitment of NER proteins to regions of stalled RNAP. Curiously though, cells lacking Mfd do not display increased sensitivity to DNA-damaging agents (Cohen et al., 2010, Epshtein et al., 2014, Kamarthapu et al., 2016, Witkin, 1966b, Witkin, 1969; Figure S1). Furthermore, overexpression of Mfd sensitizes cells to DNA damage (Kamarthapu et al., 2016). Moreover, even though Mfd is canonically known to promote DNA repair, it paradoxically increases mutagenesis in certain contexts, such as at regions of replication-transcription conflicts and in stationary-phase mutagenesis (Han et al., 2008, Lee et al., 2009, Million-Weaver et al., 2015, Martin et al., 2011, Ross et al., 2006, Wimberly et al., 2013). These findings can be interpreted in at least three different (but not mutually exclusive) ways: (1) redundant TCR mechanisms exist (also proposed by Kamarthapu et al., 2016), (2) Mfd may actually inhibit DNA repair in some contexts (also proposed by Pani and Nudler, 2017), and (3) Mfd may promote DNA repair, but this repair is mutagenic in the absence of exogenous DNA damage (e.g., Million-Weaver et al., 2015).

Mfd may have additional functions outside of TCR. Recently, Mfd was found to associate with RNAP even in the absence of exogenous DNA damage (Ho et al., 2018, Le et al., 2018), suggesting that it may play a more general housekeeping role during transcription elongation. Furthermore, Mfd acts as an RNAP anti-backtracking factor and therefore could be critical for RNAP processivity. Mfd’s anti-backtracking activity also alleviates genomic instability caused by collisions between replication and transcription elongation complexes (Dutta et al., 2011).

Here, we identify Mfd as an evolvability factor, the absence of which hinders antibiotic resistance development. We show that Mfd promotes mutagenesis in bacteria both during laboratory growth and during infection of eukaryotic cells. Our experiments show that the Mfd-dependent increase in mutagenesis accelerates AMR development and that this holds true for multiple classes of antibiotics. We also find that Mfd promotes the evolution of hypermutation, one important mechanism known to lead to rapid AMR development. Importantly, our findings show that the role of Mfd in AMR development is highly conserved across bacteria, including several clinically relevant pathogens. Finally, we pinpoint critical regions of Mfd that are required for its evolvability function. Specifically, we show that the interactions of Mfd with the RNA polymerase beta subunit RpoB as well as the NER protein UvrA are required for its role in the rapid evolution of resistance to several classes of antibiotics. Altogether, these results provide evidence that blocking evolvability factors—in particular, Mfd—can inhibit resistance development in a diverse array of bacterial pathogens.

## Results

### Mfd Is a Mutagenic Factor in Divergent Bacterial Species

**Figure 1:**
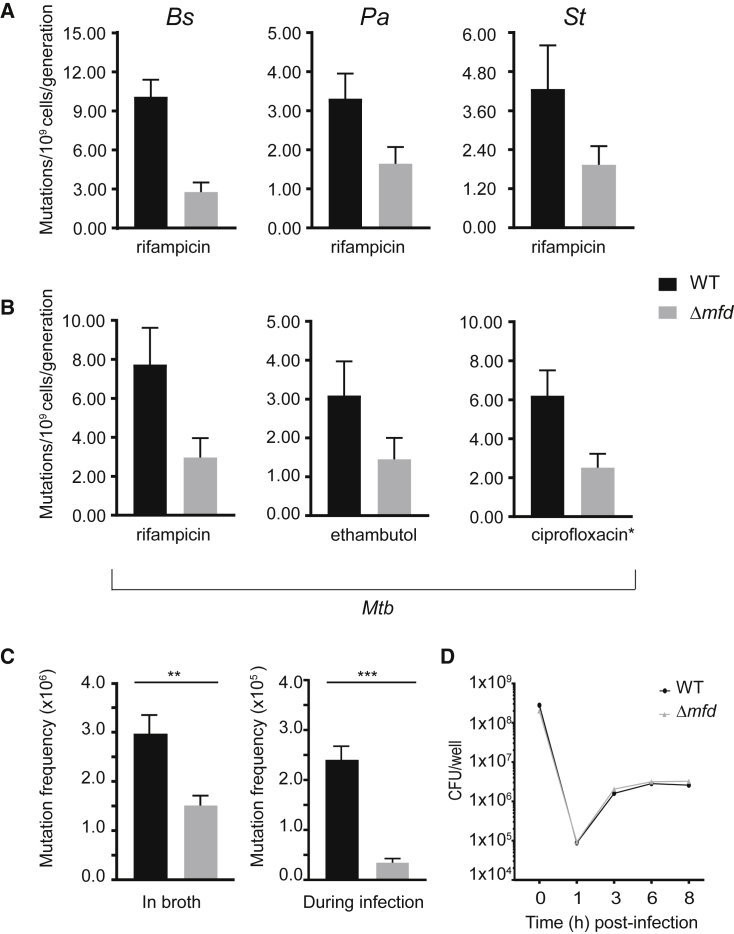
Mfd Promotes Mutagenesis in Diverse Bacterial Species, Related to Figures S1 and S5 (A) Mutation rates of WT (black) and Δ*mfd* (gray) strains to rifampicin for three indicated species (*Bs*, *B. subtilis* HM1; *Pa*, *P. aeruginosa* CF127; *St*, *S. typhimurium* ST19). Number of replicates for *Bs* = 75, *Pa* = 42, *St* = 36. Error bars are 95% confidence intervals. (B) Mutation rates of *Mtb* (H37Rv) to three different antibiotics for WT (black) and Δ*mfd* (gray). Number of replicates for *Mtb* = 33–48. Error bars are 95% confidence intervals. ^∗^Ciprofloxacin y-axis is mutations per 10^8^ cells per generation. (C) Mutation frequency of *S. typhimurium* in culture tubes and during infection of CACO-2 cells. Frequency was measured by plating on M9 glycerol with 5-flourocytosine for CFU enumeration. Error bars are standard error of the mean. Two-tailed Student’s t test determined statistical significance (^∗∗^p value < 0.01, ^∗∗∗^p value < 0.001). (D) CFU enumeration of WT and Δ*mfd S. typhimurium* strains upon infection of CACO-2 cells.

The role of Mfd in DNA repair has remained controversial: cells lacking Mfd are not sensitive to DNA-damaging agents and previous work hints at a mutagenic role for Mfd in specific contexts. We decided to thoroughly examine Mfd’s role in mutagenesis, specifically in the absence of exogenous DNA damage. We measured mutation rates with and without Mfd in divergent bacterial species using Luria-Delbrück fluctuation analysis (Luria and Delbrück, 1943). We observed that strains lacking Mfd had a 2- to 5-fold decrease in mutation rates as measured by rifampicin resistance compared to wild-type (WT) strains (Figure 1). This decreased mutation rate was conserved between Gram-negative and Gram-positive species, including *Bacillus subtilis* and clinical isolates of *Salmonella typhimurium* (Hayden et al., 2016) and *Pseudomonas aeruginosa* (Wolfgang et al., 2003; Figure 1A). These results are in contrast to Mfd’s previously published anti-mutagenic properties during UV exposure (Selby and Sancar, 1993, Witkin, 1966a).

Chromosomal mutations are the sole means by which AMR develops in *Mtb* (Almeida Da Silva and Palomino, 2011). Differences in mutation rates between clinical isolates of *Mtb* are thought to underlie AMR development (Ford et al., 2013). Therefore, if conserved, Mfd-driven mutagenesis could have a significant impact on the development of AMR in *Mtb*. Indeed, when we deleted the gene encoding Mfd, mutation rates in *Mtb* were reduced by roughly 2- to 3-fold (Figure 1B), as measured by resistance to three different antimicrobials frequently used to treat tuberculosis: rifampicin, ethambutol, and ciprofloxacin. This suggests that Mfd promotes mutagenesis across different resistance loci in *Mtb* and is likely critical for the development of antibiotic resistance.

### Mfd’s Mutagenic Function Is Conserved during Infection of Eukaryotic Cells

We wanted to determine if the mutagenic effects of Mfd are conserved in an infection model of drug resistance. For these experiments, we infected CACO-2 epithelial cells with a clinical isolate of *S. typhimurium* and subsequently measured mutation frequency using resistance to 5-fluorocytosine (Richardson et al., 2009). Interestingly, compared to the ∼2- to 4-fold decrease observed during laboratory growth (Figure 1C, left), we see a ∼5-fold decrease in mutagenesis in the absence of Mfd upon host cell infection (Figure 1C, right). These differences are not related to growth defects during infection of host cells, as there is no change in the number of colony-forming units following infection (Figure 1D). Therefore, the effect of Mfd-mediated mutagenesis is both conserved and potentially enhanced during growth and replication in the host.

### Mfd Accelerates AMR Development

**Figure 2:**
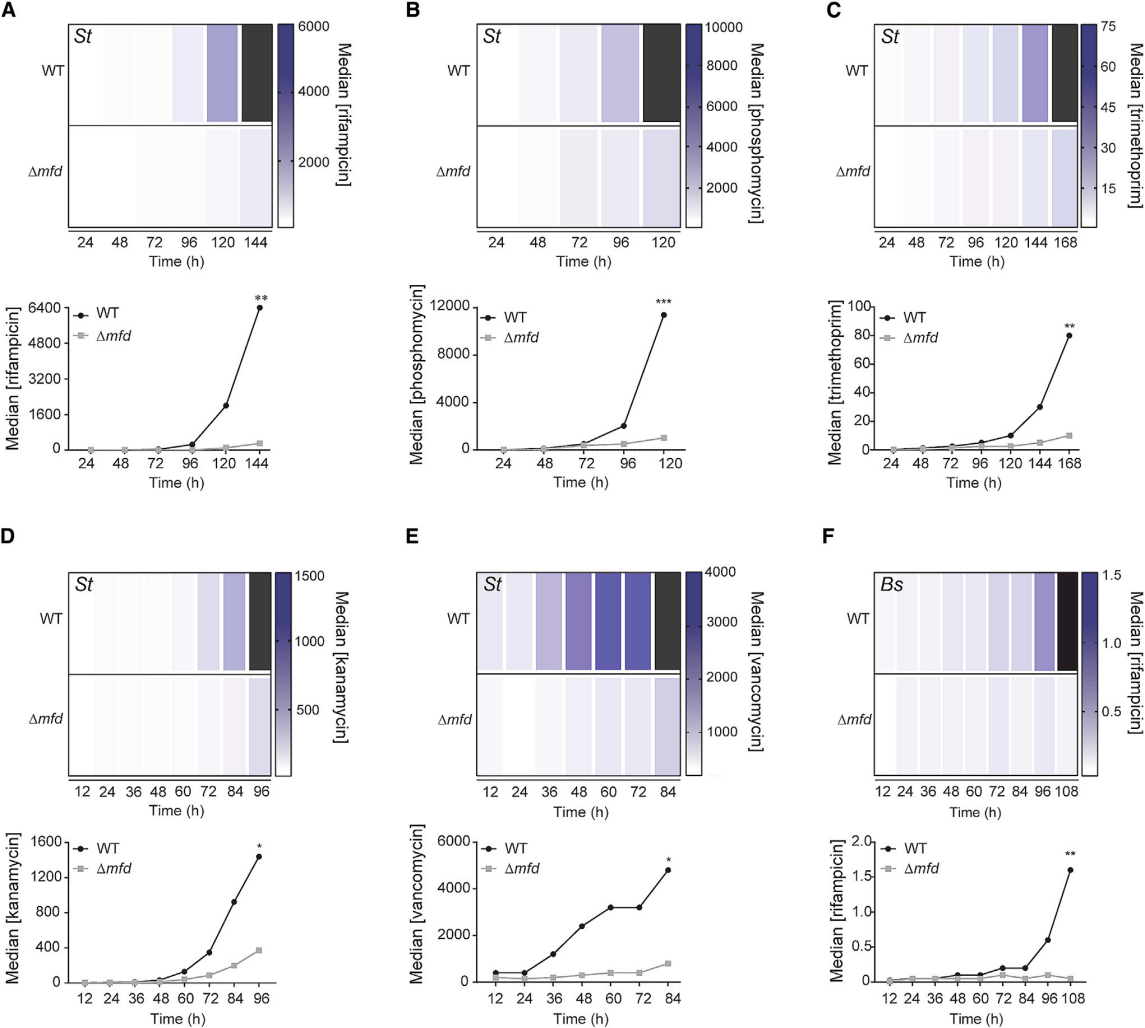
Mfd Promotes Evolution to Various Classes of Antibiotics, Related to Figure S2, S3, and Table S1 Evolution of *S. typhimurium* ST19 to (A) rifampicin, (B) phosphomycin, (C) trimethoprim, (D) kanamycin, and (E) vancomycin; evolution of *B. subtilis* HM1 to (F) rifampicin. Heatmaps and line plots show median antibiotic concentration for WT and Δ*mfd* strains at each sampled time point. Black bars represent median growth greater than highest concentration shown on the scale. Concentrations for all antibiotics are in μg/mL. Statistical significance was determined using a two-tailed Mann-Whitney U test (^∗^p value < 0.05, ^∗∗^p value < 0.01, ^∗∗∗^p value < 0.001). Number of replicates for each strain and antibiotic of *St* and *Bs* are 12–30.

We next assessed the impact of Mfd on both the kinetics and the levels of AMR development in short-term evolution experiments in the Gram-negative pathogen *S. typhimurium*. Given that the differences in mutation rates between WT and cells lacking Mfd were modest (2- to 5-fold), we wondered if these differences could impact the kinetics and evolution of resistance in a meaningful way. To test this model, we developed an assay that measures both metrics over roughly 35 to 70 generations in the presence of antibiotics (ranging from sub-inhibitory to ∼16x MIC for our first time course). We monitored the evolution of *S. typhimurium* resistance to a panel of clinically relevant antibiotics (rifampicin, phosphomycin, trimethoprim, kanamycin, and vancomycin), which act through different mechanisms and have different resistance loci. We found that resistance to all the antibiotics tested arose significantly faster and to higher levels in WT compared to the Δ*mfd* strain (Figures 2A–2E). The difference in the median resistance levels between the two strains at the end of the *S. typhimurium* evolution experiments was 6-to 21-fold greater in WT than in Δ*mfd*. These results were not specific to *S. typhimurium*: we found a ∼32 fold difference in median antibiotic concentration tolerated by WT compared to Δ*mfd* cells in the highly divergent, Gram-positive bacterium *B. subtilis* (Figure 2F). These findings show that Mfd promotes resistance development in diverse bacterial species.

### Mfd Is Critical for the Development of AMR in *Mtb*

**Figure 3:**
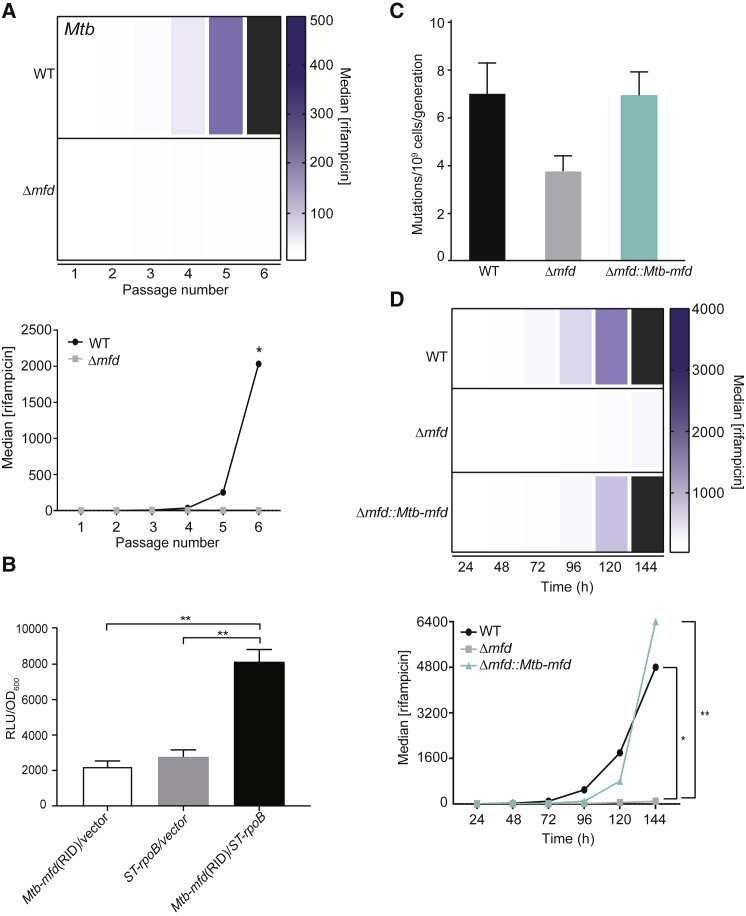
Mfd Promotes Evolution to Antibiotics in *Mtb* (A) Evolution of *Mtb* H37Rv to rifampicin. Heatmaps and line plots showing median rifampicin concentration for WT and Δ*mfd* strains at each sampled passage from a representative experiment are shown. Black bars represent median growth greater than highest concentration shown on the scale. Concentrations are in ng/mL. Statistical significance was determined using a two-tailed Mann-Whitney U test (^∗^p value < 0.10). Number of replicates for each strain of *Mtb* is 6. (B) *Mtb* Mfd and ST19 RpoB interact. *Mtb* Mfd RNAP interacting domain (RID) and *S. typhimurium* ST19 RpoB N-terminal domain were cloned into a luciferase-based bacterial 2-hybrid system. Interactions between these respective protein domains were measured by luminescence and normalized to OD_600_. Results are from three independent experiments, and error bars indicate standard error of the mean. Statistical significance was determined using two-tailed Student’s t test (^∗∗^p value < 0.01). (C) Mutation rate analyses were performed with indicated strains of *S. typhimurium* to rifampicin as in Figure 1. Number of replicates is 36–96. (D) Evolution of indicated strains of *S. typhimurium* to rifampicin. Plots and statistical testing for evolution assays were performed as described in Figure 2. Number of replicates per strain is 12–24. ^∗^p value < 0.05 between WT and Δ*mfd* strains and ^∗∗^p value < 0.01 between Δ*mfd::Mtb-mfd* and Δ*mfd* strains.

*Mtb* is arguably the most difficult-to-treat pathogen due to AMR development. Therefore, we were interested in determining whether Mfd is responsible for the evolution of resistance in this pathogen. We adapted our short-term evolution assays to the unique culture conditions of *Mtb* and performed the evolution experiments using rifampicin as a representative antibiotic. The difference in median resistance to rifampicin between the two strains at the end of the experiment was striking: the median resistance level to rifampicin in WT was in some experiments up to 1,000-fold greater than Δ*mfd* strains (Figure 3A). This difference is significantly greater than that observed for *S. typhimurium* or *B. subtilis*. Additionally, we find that by the end of our evolution assays, roughly 2/3 of our evolved WT strains were above the clinical MIC breakpoint of *Mtb* to rifampicin (1 mg/L) (Schon et al., 2009), whereas none of the Δ*mfd* strains reached this threshold. These data suggest that, as observed in other species, Mfd is critical in the development of AMR in *Mtb*—a finding with potential clinical implications.

### The Evolvability Function of Mfd Can Be Cross-complemented between Divergent Species

The data presented above suggest that Mfd’s role in AMR development is conserved across species. To test the degree of conservation, we first performed bacterial 2-hybrid assays to determine if Mfd’s well-documented interaction with RpoB can be detected between *S. typhimurium* and *Mtb* proteins. We chose to test these species for our experiments because they are highly divergent. Furthermore, although minimal, these two species have the biggest difference in the Mfd sequence at the amino acid level. We found that *S. typhimurium* RpoB interacts with the *Mtb* Mfd-RNAP interaction domain (RID) (Figure 3B). These data suggest that the mechanism by which Mfd promotes the evolution of AMR could be conserved across these species.

We then performed cross-complementation experiments using *S. typhimurium* and *Mtb* as models (Figures 3C and 3D). We introduced a copy of the *Mtb mfd* gene into *S. typhimurium* strains lacking *mfd* and performed both mutation rate and evolution assays to rifampicin. Strikingly, the *Mtb mfd* gene fully complemented the reduced mutation rates (Figure 3C) as well as the delayed evolution of Δ*mfd S. typhimurium* resistance to rifampicin (Figure 3D). These results indicate that the mechanism facilitating the evolvability function of Mfd is highly conserved across bacterial species.

### Mfd Promotes Evolution by Increasing Mutagenesis

To determine if Mfd promotes AMR development through its mutagenic properties, we used Sanger sequencing to identify mutations that arose within the known rifampicin and trimethoprim resistance genes (*rpoB* [Brandis et al., 2015] and *folA* [Watson et al., 2007], respectively) during our evolution experiments. Analysis of the sequences obtained from every time point for 12 different replicates in both *S. typhimurium* WT and Δ*mfd* strains revealed several resistance mutations. However, the Δ*mfd* replicates consistently accumulated roughly 1/2 of the number of mutations in *rpoB* and 1/3 of the number of mutations in *folA* compared to WT (Figures S2A and S2B). Importantly, we observed a significant delay in the acquisition of mutations in the Δ*mfd* strains compared to those in WT and rarely observed additional second and third mutations in the Δ*mfd* strains (Figures S2A and S2B). These data strongly suggest that Mfd promotes the evolution of resistance to antibiotics through its pro-mutagenic function and that it may be critical for the acquisition of multiple mutations.

### Mfd Promotes the Rise of Hypermutators

To determine if there were any mutations outside of the resistance loci in WT compared to Δ*mfd* strains, we performed whole-genome sequencing (WGS) of six randomly chosen replicates from our rifampicin and trimethoprim evolution experiments. WGS of six WT and Δ*mfd S. typhimurium* isolates from every time point of our rifampicin and trimethoprim evolution assays confirmed that, compared to our WT strain, Δ*mfd* strains accumulated significantly fewer mutations in the *rpoB* locus, as we had observed using Sanger sequencing (Figures S2A and S2B). We did not find any additional mutations outside of the *rpoB* gene in any of the evolved rifampicin-resistant strains that we sequenced. In contrast, our WGS of strains evolved in trimethoprim revealed the presence of additional mutations outside of the coding region, within the putative promoter region of *folA*. All six sequenced WT strains contained one of two putative promoter mutations (either 35 or 61 base pairs upstream of the *folA* coding sequence), while only one of our sequenced Δ*mfd* isolates carried one of these mutations (Table S1).

Interestingly, we found that three out of six WT sequenced trimethoprim-evolved strains contained a point mutation in the *dnaQ* gene (all strains had the same *dnaQ*(I33N) mutation), while none of the Δ*mfd* strains contained any mutations in the *dnaQ* gene (Table S1). Mutations in *dnaQ* are known to generate hypermutator phenotypes (Echols et al., 1983), so, to determine if this new allele indeed conferred a hypermutator phenotype, we performed Luria-Delbrück fluctuation analysis of an evolved WT strain before and after gaining the identified *dnaQ* mutation. We found that the mutation rate upon gaining this *dnaQ* allele was ∼1,000-fold higher than the ancestor strain (Figure S3). We subsequently performed Sanger sequencing of the *dnaQ* allele on four additional WT and Δ*mfd* strains and found that two out of four WT strains contained the same *dnaQ* mutation, while none of the four Δ*mfd* strains contained this mutation. Overall, we can estimate that roughly 50% of WT strains developed hypermutator alleles during the evolution of trimethoprim resistance, while strains lacking Mfd are restrained in developing this phenotype (we did not find a hypermutator Δ*mfd* isolate).

As expected, we found that WT *S. typhimurium* isolates carrying the *dnaQ* hypermutator allele accumulated a high number of mutations across the genome (up to 600 in some of our evolved isolates), including mutations that may confer an adaptive advantage in the presence of trimethoprim. These mutations should be examined further to discern true adaptive mutations from hitchhiker mutations. These potentially adaptive mutations are in genes previously implicated in promoting trimethoprim resistance (Baym et al., 2016), such as *aroK* (involved in the shimikate pathway for folate synthesis) (Table S1). We also found putative adaptive mutations in genes not previously associated with trimethoprim resistance. These mutations arose in genes such as *fis* (DNA binding and regulator of replication initiation and global transcription), *pyrG* (CTP synthetase), *ygdP* (RNA pyrophosphohydrolase), and *ybgC* (Acyl-CoA thioester hydrolase), among many others (Table S1). Mutations in many of these genes arose in independent lineages, suggesting that they may confer adaption to trimethoprim. These findings show that, in our evolution assays (as in clinical settings), the generation of hypermutation may offer an adaptive strategy to evolve high-level antibiotic resistance and that Mfd promotes this phenomenon.

### Mfd-Mediated Evolution Requires Its Interaction with RNAP and UvrA

**Figure 4:**
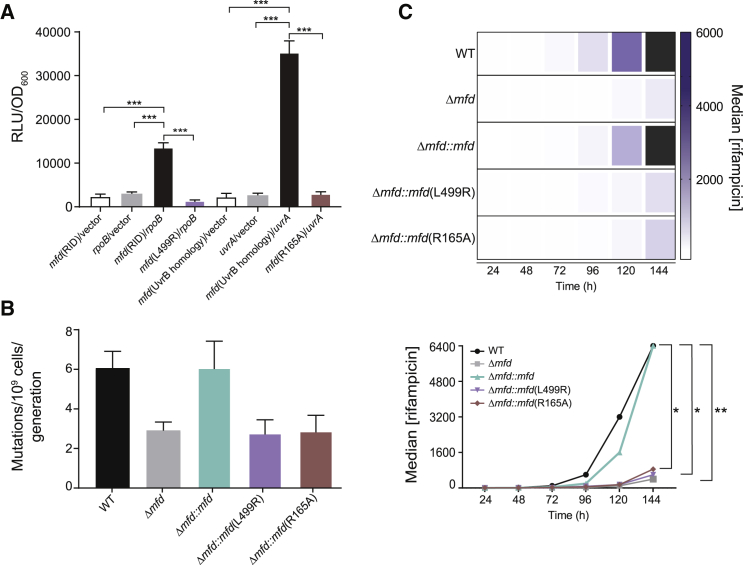
Mfd-RpoB and Mfd-UvrA Interactions Are Essential for Mfd-Driven Mutagenesis and Evolution to Antibiotics, Related to Figure S4 (A) Mutation of Mfd L499R and R165A residues abrogates RNAP and UvrA interactions, respectively. Relevant domains of the RpoB, Mfd, and UvrA proteins of *S. typhimurium* ST19 were cloned into a luciferase-based bacterial 2-hybrid system. Interactions between the respective protein domains were measured as in Figure 3. Results are from three independent experiments, and error bars indicate standard error of the mean. Statistical significance was determined using two-tailed Student’s t test (^∗∗∗^p value < 0.001). (B) Mutation rate analysis of indicated strains of *S. typhimurium* to rifampicin. Complement and point mutant (L499R and R165A) strains were expressed episomally. WT and Δ*mfd* strains contain pUC19 empty vector control (see Tables S2 and S3). Number of replicates per strain is 36–112. Errors bars are 95% CI. (C) Evolution of indicated *S. typhimurium* strains to rifampicin. Complement and point mutant strains (L499R and R165A) were expressed episomally. WT and Δ*mfd* strains contain pMMB67EH empty vector controls. Strains were grown in 50 μg/mL carbenicillin to maintain selection of episomes. Plots and statistical testing for evolution assays were performed as described in Figure 2. Number of replicates per strain is 12–24. ^∗∗^p value < 0.01 between WT and Δ*mfd* strains and ^∗^p value < 0.05 between WT and Δ*mfd::mfd*(L499R) and WT and Δ*mfd::mfd*(R165A) strains.

To test whether the evolvability function of Mfd depended on transcription and its conserved interaction with RpoB, we constructed an L499R mutation in the RNAP interaction domain (RID) of *S. typhimurium* Mfd. The Mfd L499R mutant was previously characterized in *E. coli* and was shown to alter Mfd-RpoB interaction without affecting Mfd’s DNA binding and ATPase activity (Deaconescu et al., 2006). Our bacterial 2-hybrid assays confirmed that disrupting this residue in *S. typhimurium* Mfd abrogates its binding to RpoB (Figure 4A). We found that WT Mfd fully complements the decreased evolvability of Δ*mfd* strains, whereas the L499R point mutant cannot complement the evolution of resistance to rifampicin (Figures 4B and 4C), trimethoprim, or phosphomycin (Figure S4). Therefore, the interaction between Mfd and RpoB is essential for Mfd’s mutagenesis and subsequent evolvability function.

We next sought to determine whether Mfd’s interaction with its other known binding partner, UvrA, was also necessary for its evolvability function. In order to test this, we constructed a point mutation in the D2 domain (part of the UvrB homology module) of the Mfd protein, known to mediate binding to UvrA (Deaconescu et al., 2006). Bacterial 2-hybrid assays confirmed that a point mutation in the UvrA interaction domain of *S. typhimurium* Mfd (R165A) successfully abrogated its binding to UvrA (Figure 4A). We expressed *S. typhimurium* Mfd containing the R165A point mutation in the Δ*mfd* background and tested the impact of this mutant on mutation rates and evolution of resistance to rifampicin or phosphomycin. The Mfd R165A mutant was unable to complement any of the phenotypes (lowered mutagenesis or restricted evolution of resistance) associated with the lack of Mfd (Figures 4B–4C and S4A). The D2 domain of Mfd is also thought to form an interface with the C terminal D7 domain as a means of restricting Mfd-UvrA interactions in the absence of RNAP stalling (Deaconescu et al., 2006). While we cannot exclude the possibility that the R165A mutation alters this interaction, it is unlikely that we can attribute a disruption of the interface between D2 and D7 to our observed results. The major effects of abrogating D7 activity are thought to be tighter binding of Mfd to UvrA (Deaconescu et al., 2006, Savery, 2007, Selby and Sancar, 1995), yet the phenotypes of this mutant mimic those of Δ*mfd* strains. Overall, our data suggest that Mfd promotes mutagenesis and evolution of drug resistance through its interactions with both RpoB and UvrA.

## Discussion

In this work, we assign a novel function to Mfd as a general evolvability factor and demonstrate that it accelerates AMR development. We show that Mfd’s evolvability function requires its evolutionarily conserved interactions with both RpoB and UvrA. Arguably, the ability to evolve is critical for bacterial survival under ever-changing environmental conditions. This is especially important in the context of pathogenesis, where escaping host immunity is essential and requires constant adaptation. Therefore, our model of Mfd as an evolvability factor could explain its high degree of conservation across phyla, especially in pathogens, which are unlikely to experience TCR activation through UV exposure. This model is consistent with data showing that a deletion of Mfd has minor effects on DNA damage tolerance—especially compared to cells missing NER (Figure S1; Witkin, 1966b, Witkin, 1969)—and that bacteria lacking Mfd are insensitive to the DNA-damaging environment within macrophages (Figure S5). These observations suggest that Mfd is largely dispensable for the coordination of DNA repair. TCR is still an important and effective method of lesion detection and repair; however, Mfd may not be the main driver of this DNA repair mechanism. Recently, a new TCR pathway (Cohen et al., 2010) driven by the helicase UvrD (Epshtein et al., 2014, Kamarthapu et al., 2016) was discovered, indicating that cells also harbor Mfd-independent TCR mechanisms.

How Mfd promotes antimicrobial resistance and mutagenesis is unclear. One possible explanation is that Mfd promotes mutagenic DNA repair through error-prone gap filling at sites of NER activity, as previously suggested (Million-Weaver et al., 2015). Mfd may also promote DNA repair at sites that do not contain damaged DNA, given that Mfd can associate with RNAP in the absence of exogenous DNA damage (Ho et al., 2018). *In vitro* data showing that NER can promote gratuitous repair of undamaged DNA leading to recurrent DNA re-synthesis, which could consequently promote mutagenesis, is also consistent with this model (Branum et al., 2001). Alternatively, Mfd may promote mutagenesis by inhibiting the activation of other DNA repair pathways, at least under “normal” growth conditions—e.g., absence of UV damage. These pathways may include Mfd-independent TCR or global NER. Given our data with the UvrA interaction mutant of Mfd, we would predict that such inhibition would be through sequestration of UvrA.

Mfd-mediated evolution may be critical in the context of host infection. During infections, bacterial replication is reduced (Gill et al., 2009, Helaine et al., 2010), consequently reducing replication fork errors and possibly enhancing the relative contribution of non-replicative mutations (Gao et al., 2016). Given that transcription is still active under these conditions, Mfd may play a critical role in promoting bacterial mutagenesis during infections. This may explain the exaggerated effects of Mfd that we observed in our infection model. Additionally, our evolution assays, which mimic the variable antibiotic concentrations seen during clinical infections, suggest that Mfd is required for developing high levels of drug resistance upon primary exposure to sub-inhibitory concentrations of antibiotics, which may be critical in the context of AMR development (Wistrand-Yuen et al., 2018).

Lastly, Mfd may be even more important when multiple mutations are necessary to confer resistance, such as in the context of multi-drug resistance acquisition or in the context of compensatory mutations. Our sequencing data are consistent with this prediction, given that the occurrence of second and third mutations was quite rare in the absence of Mfd. Additionally, we observe that the rise of *dnaQ* hypermutator strains is inhibited in the absence of Mfd. Hypermutation is a key strategy that bacteria use to evolve resistance in the context of infections (Blázquez, 2003, Oliver et al., 2000). However, strains specifically containing the *dnaQ* hypermutator alleles have not been identified in clinical settings. This could simply be due to the fact that few (if any) isolates from trimethoprim-treated patients have been sequenced. Therefore, it is still possible that the *dnaQ* hypermutator allele is relevant in clinical settings. This question should be investigated by WGS of pathogens isolated from trimethoprim-treated patients.

Given our findings, we propose that blocking evolvability factors, and in particular Mfd, could be a revolutionary strategy to address the AMR crisis. A new class of “anti-evolution” drugs that target Mfd or other evolvability factors that promote mutagenesis may complement new antimicrobials and alleviate the problem of chromosomally acquired mutations that promote AMR. For example, LexA, which induces the SOS response upon exposure to DNA damage, has been suggested to promote AMR development, likely through trans-lesion synthesis (TLS) at replication forks (Cirz et al., 2005, Cirz et al., 2006, Mo et al., 2016). This mechanism could also be a good target for the inhibition of AMR development. However, SOS-mediated AMR development may be distinct from the transcription-dependent evolvability function of Mfd, which (in addition to replicating cells) could be relevant in infections in which pathogens are not replicating and/or have not been exposed to extensive DNA damage but are transcriptionally active. Therefore, in principle, drugs that target Mfd (or key SOS factors) could be co-administered with antibiotics during treatment of infections, reducing the likelihood of resistance development at the onset of treatment. Overall, efforts to understand and target the evolutionary capacity of cells could also have wide-ranging implications outside of AMR development, from reducing cancer evolution to limiting pathogenic diversity in the context of host immunity.

The ideas discussed here deliver a second message regarding drug discovery and therapeutics. Although drug discovery efforts are generally geared toward targeting essential proteins, the effectiveness of this approach may be limited. Supplemental drugs that target non-essential proteins (e.g., Mfd) during the treatment of infections (or various diseases such as cancer) have the potential to significantly improve the efficiency and/or potency of current treatment regimens. Therefore, development of novel therapeutics targeting non-essential proteins could expand the arsenal of drugs available to combat AMR and potentially other diseases.

## STAR★Methods

### Key Resources Table

**Table:** 

REAGENT or RESOURCE	SOURCE	IDENTIFIER
**Chemicals, Peptides, and Recombinant Proteins**		

BamHI-HF	NEB	R3136S
HindIII-HF	NEB	R3104S
NotI-HF	NEB	R3189S
BglII	NEB	R0144S
NheI	NEB	R0131S
XhoI	NEB	R0146S
4-Nitroquinoline N-oxide	Sigma	N8141
Gibson Assembly Master Mix	NEB	E2611S
Phusion High-Fidelity PCR Master Mix	Thermo	F531S
Nano-Glo Luciferase Assay System	Promega	N1130

**Critical Commercial Assays**		

Nextera XT DNA Library Preparation Kit	Illumina	(FC-131-1024)
GeneJET PCR Purification Kit	Thermo	K0701
GeneJET Genomic DNA purification Kit	Thermo	K0722
MasterPure complete DNA and RNA Purification Kit	Epicenter	MC85200
QIAquick PCR Purification Kit	QIAGEN	28106

**Deposited Data**		

All fastq files and descriptions uploaded to Sequence Read Archive	NCBI SRA SUB4542953	Bioproject: PRJNA492467

**Experimental Models: Cell Lines**		

Human: Caco-2 epithelial cell line	ATCC	HTB-37; RRID:CVCL_0025
Harvested Bone Marrow Macrophages from BALB/c mice	N/A	Miller Lab

**Experimental Models: Organisms/Strains**		

*B. subtilis trpC2 pheA1*	Brehm et al., 1973	HM1
*B. subtilis trpC2 uvrA::mls*	Bacillus stock center	HM713
*B. subtilis trpC2 mfd::mls*	Bacillus stock center	HM1720
*S. typhimurium* ST19	Hayden et al., 2016	HM1996
*P. aeruginosa* CF127	Wolfgang et al., 2003	HM2212
*P. aeruginosa* CF127 Δ*mfd*	This study	HM2260
*E. coli F’(kan)* placOL2-62-lacZ	Dove et al., 1997	HM2295
*B. subtilis mfd::mls, trpC2 pheA1*	Million-Weaver et al., 2015	HM2521
*E. coli F’(kan)* placOL2-62, pSIM27(tet)	This study	HM2602
*B. subtilis uvrA::mls, trpC2 pheA1*	Million-Weaver et al., 2015	HM2633
*E. coli* FW102 OL2-62-Nanoluc(hyg), pSIM27	This study	HM2747
*E. coli* FW102 OL2-62-Nanoluc(hyg), pACλCI, pBRα Plac-a-ST19rpoB(19-142)	This study	HM2838
*E. coli* FW102 OL2-62-Nanoluc(hyg), pBRα, pACλCI Plac-CI-ST19mfd(1-450)	This study	HM2875
*S. typhimurium* ST19, pUC19	This study	HM2880
*S. typhimurium* ST19 *mfd::cat*, pUC19	This study	HM2881
*S. typhimurium* ST19 *mfd::cat*, pUC19-ST19mfd	This study	HM2882
*S. typhimurium* ST19 *mfd::cat*, pUC19-ST19mfdL499R	This study	HM2886
*E. coli* FW102 OL2-62-Nanoluc(hyg), pACλCI Plac-CI-ST19mfd(1-450), pBRα Plac-a-ST19UvrA(88-505)	This study	HM2913
*E. coli* FW102 OL2-62-Nanoluc(hyg), pACλCI Plac-CI-ST19rpoB(19-142), pBRα Plac-a-ST19mfd (RID)	This study	HM2920
*E. coli* FW102 OL2-62-Nanoluc(hyg), pACλCI Plac-CI-ST19rpoB(19-142), pBRα Plac-a-ST19mfd (RID) L499R	This study	HM2921
*E. coli* FW102 OL2-62-Nanoluc(hyg), pACλCI Plac-CI-ST19rpoB(19-142), pBRα	This study	HM2925
*E. coli* FW102 OL2-62-Nanoluc(hyg), pACλCI, pBRα Plac-a-ST19mfd (RID)	This study	HM2926
*E. coli* FW102 OL2-62-Nanoluc(hyg), pACλCI, pBRα Plac-a-ST19UvrA(88-505)	This study	HM2949
*E. coli* FW102 OL2-62-Nanoluc(hyg), pACλCI Plac-CI-ST19mfdR165A, pBRα Plac-a-ST19UvrA(88-505)	This study	HM2962
*S. typhimurium* ST19 *mfd::cat*, pUC19-Mtbmfd	This study	HM3134
*E. coli* FW102 OL2-62-Nanoluc(hyg), pACλCI Plac-CI-Mtbmfd(334-651), pBRα	This study	HM3193
*E. coli* FW102 OL2-62-Nanoluc(hyg), pACλCI Plac-CI-Mtbmfd(334-651), pBRα Plac-a-ST19rpoB(19-142)	This study	HM3225
*S. typhimurium* ST19 *uvrA::kan*	This study	HM3245
*S. typhimurium* ST19 ST19*mfd::Mtbmfd-kan*	This study	HM3406
*S. typhimurium* ST19 *mfd::cat*	This study	HM3429
*S. typhimurium* ST19, pMMB67EH	This study	HM3585
*S. typhimurium* ST19 *mfd::cat*, pMMB67EH	This study	HM3586
*S. typhimurium* ST19 *mfd::cat*, pMMB67EH-ST19mfd	This study	HM3590
*S. typhimurium* ST19 *mfd::cat*, pMMB67EH-ST19mfdR165A	This study	HM3666
*S. typhimurium* ST19 *mfd::cat*, pMMB67EH-ST19mfdL499R	This study	HM3667
*M. tuberculosis* H37Rv	ATCC	ATCC 27294
*M. tuberculosis* H37Rv mfd::hyg	This study	MR02

**Oligonucleotides**		

Primer sequences provided in Table S3	N/A	N/A

**Recombinant DNA**		

pHM443	This study	pHM443
pHM453	This study	pHM453
pHM457	This study	pHM457
pHM458	This study	pHM458
pHM474	This study	pHM474
pHM480	This study	pHM480
pHM481	This study	pHM481
pHM484	This study	pHM484
pHM494	This study	pHM494
pHM499	This study	pHM499
pHM550	This study	pHM550
pHM566	This study	pHM566
pHM629	This study	pHM629
pHM649	This study	pHM649
pHM650	This study	pHM650
pHM651	This study	pHM651
pHM661	This study	pHM661
pHM662	This study	pHM662
pBRα	Addgene	Addgene 53731
pBRα-β-flap	Addgene	Addgene 53734
pACλCI	Addgene	Addgene 53730
pACλCI-β-flap	Addgene	Addgene 53733
pEX18	Gift from Dr. Matthew Parsek	pEX18
pKD3	Addgene	Addgene 45604
pKD13	Miller Lab Stock	pKD13
pKD46	Miller Lab Stock	pKD46
pMMBEH67	Miller Lab Stock	pMMBEH67
pNIT	Sherman Lab Stock	pNIT
pNL1.1	Promega	Promega N1441
pSIM27	Gift from Dr. Don Court	pSIM27
pUC19	Addgene	Addgene 50005

**Software and Algorithms**		

SAMtools	Li et al., 2009	N/A
Bowtie 2	Langmead and Salzberg, 2012	N/A
breseq	Deatherage and Barrick, 2014	N/A
Prism 7 Graphpad	N/A	N/A

### Contact for Reagent and Resource Sharing

Further information and requests for resources and reagents should be directed to Lead Contact, Houra Merrikh (merrikh@uw.edu).

### Experimental Model and Subject Details

Strains for the following species were built as described: deletions in *B. subtilis* were built with transformation of marked genomic DNA into the appropriate background strain. Deletions in *S. typhimurium* were built using the λ-red recombineering (Datsenko and Wanner, 2000) and all plasmids used were transformed by electroporation. The *mfd* deletion in *P. aeruginosa* was built using the pEX18 suicide plasmid (with homology regions to *mfd*) as previously described (Hoang et al., 1998), and the *mfd* deletion in *Mtb* was built using recombineering (van Kessel and Hatfull, 2007). *E. coli* strains for the bacterial 2-hybrid assay were built via electroporation of the designated plasmid into the appropriate strain background. *E. coli* DH5α was used to propagate recombinant DNA vectors. Transformations were done using heat shock of competent *E. coli*. *E. coli* cultures were grown at 37°C with shaking (260 RPM) in LB supplemented with antibiotics where appropriate. All plasmid vectors were purified using a commercially available plasmid extraction kit (Thermo). Specific details of strains and plasmid constructed used in this work, including primers used, are listed in Table S2 and S3. All strain modifications were confirmed by PCR and sequencing.

### Method details

#### Strain constructions

Details of bacterial strains and recombinant plasmids built in this study are described in Table S2.

#### Luria-Delbruck fluctuation analysis

For *B. subtilis*, cultures were grown from single colonies at 37°C with aeration in LB media (10 g Tryptone, 5g yeast extract and 5g NaCl per liter). Exponential phase cultures (OD_600_ = 0.3) were diluted back to OD_600_ = 0.0005 in parallel cultures containing LB, and plated following 4.5 hours of growth at 37°C with aeration. Cultures were plated on 50 μg/mL rifampicin to quantify the number of mutants and serially diluted and plated on LB to quantify total viable cells. Colonies were quantified after overnight incubation at 37°C (for rifampicin plates) and 30°C (for LB plates).

*S. typhimurium* and *P. aeruginosa* mutation rates were measured by growing overnight cultures from single colonies and subsequently back diluting parallel cultures to an OD_600_ = 0.0005 in LB. Cultures were grown to an OD_600_ = 0.8-1.0 (OD_600_ = 1.0 for *P. aeruginosa*) at 37°C with aeration. Cultures were plated as described for *B. subtilis*. For mutation rate analysis of WT-pUC19, Δ*mfd*-pUC19, ST19 *mfd* complementation, *Mtb mfd* complementation, and the ST19 *mfd* point mutant (L499R and R165A) strains of *S. typhimurium*, overnight cultures were grown to saturation in 50 μg/mL carbenicillin to maintain plasmid selection. Cultures were back diluted to an OD_600_ = 0.0005, grown in LB only to OD_600_ = 0.8-1.0 and plated as previously described.

For *Mtb*, experiments were performed as previously described (Ford et al., 2013). Briefly, cultures were grown in 7H9 mycobacterial media + ADC to saturation. Multiple, independent cultures were back diluted to final OD_600_ = 0.0001 and grown at 37°C to OD_600_ = 0.8-1.2. Cells were plated on 7H10 mycobacterial agar + OADC and 2μg/mL rifampicin, 5μg/mL ethambutol or 1.5μg/mL of ciprofloxacin to quantify resistant mutants and on 7H10 + OADC for CFU enumeration. Plates were incubated at 37°C for approximately 10 days for CFU enumeration and 25-30 days for antibiotic plates. Mutation rates for all species were calculated using the Ma-Sandri-Sarkar Maximum Likelihood method (Hall et al., 2009).

#### Mutagenesis measurements post epithelial cell infection

Colorectal adenocarcinoma cells line CACO-2 were cultured in DMEM medium with 20% heat-inactivated FBS at 37°C in 5% CO_2_. Approximately 10^6^ CACO-2 cells were plated overnight in 6-well plates at 37°C in 5% CO_2_ for infection. A single *S. typhimurium* colony was picked and grown overnight at 37°C in LB, diluted back to an OD_600_ = 0.05 the following day and grown at 37°C in LB until cultures reached OD_600_ = 0.5. Cells were washed 2x with 1X PBS resuspended in DMEM +20% FBS and inoculated at 100:1 multiplicity of infection with CACO-2 cells at 37°C in 5% CO_2_ for one hour. Cells were then washed 2x with 1X PBS and DMEM + 20% FBS + 50 μg/mL gentamicin was added to plates to kill extracellular bacteria. After 6 hours of infection, cells were washed in 1X PBS and lysed in 1X PBS + 0.1% Triton X-100. Cells were plated on M9 minimal + 0.4% glycerol agar for CFU enumeration and M9 minimal + 0.4% glycerol agar containing 100μg/mL 5-fluorocytosine (5FC) and grown at 37°C to determine mutation frequency. Mutation frequency was determined by taking the ratio of 5FC colonies to the viable cell count for each sample. For experiments measuring cell viability over multiple time points, S. typhimurium and CACO-2 cells were grown as described and bacterial cells were harvested for CFU enumeration at defined time points.

#### Antibiotic evolution assays

Evolution experiments were performed for the indicated strains. For *S. typhimurium*, overnight cultures, started from a single colony, were back diluted to OD_600_ = 0.005 and used to inoculate a 96-well plate. Cells were grown for either 12 or 24 hours with agitation, at 37°C, in LB with a gradient of concentrations of the indicated antibiotic to select for resistance. ODs were subsequently measured in an Epoch/2 microplate spectrophotometer (Bio-Tek). Cultures that grew (defined by at least 50% growth relative to LB only) at the highest concentration of antibiotic were passaged into fresh LB +antibiotic in a subsequent plate. A total of 5-8 serial passages were performed depending on the antibiotic used. Evolution experiments with WT-pMMB67EH, Δ*mfd* –pMMB67EH, complementation and point mutant (L499R and R165) strains of *S. typhimurium* were grown identical to WT and Δ*mfd* strains except with the addition of 50 μg/mL carbenicillin to maintain selection of episomes. For *B. subtilis*, cultures were started from a single colony were grown for 4-5 hours until they reached OD_600_ = 1.0. Cultures were back diluted to OD_600_ = 0.005, inoculated into a 96-well plate and grown for 12 hours at 37°C in LB in an Epoch/2 microplate spectrophotometer (Bio-Tek) for 9 serial passages, with a gradient of concentrations of rifampicin to select for resistance. For *Mtb*, saturated cultures were back diluted to OD_600_ = 0.05, inoculated into a 96-well plate and grown in 7H9 +ADC in a 37°C incubator without aeration. Strains were serially passaged when the density of no antibiotic control wells reached approximately OD_600_ = 1.5-2.0 (approximately 15-20 days). Cultures that grew (defined by at least 50% growth relative to 7H9+ADC) at the highest concentration of rifampicin were passaged into a fresh 7H9 + ADC+ rifampicin in a subsequent plate, and a total of 6 serial passages were performed. For all species, antibiotics were diluted 2-fold down each given row in a 96 well plate.

#### Sequencing of antibiotic evolution assays

Genomic DNA was harvested from evolved strains of *S. typhimurium* and purified using either the MasterPure complete DNA and RNA Purification Kit (Epicenter) or GeneJet Genomic DNA Purification Kit (ThermoFisher) in accordance with manufacturer instructions. For WGS experiments, gDNA samples were processed for sequencing using the Nextera XT DNA Library Preparation Kit (Illumina). Paired-end libraries were sequenced on an Illumina NextSeq sequencing platform yielding an average of 40X coverage sequencing depth. The resulting FASTQ reads were trimmed for quality using the FASTX quality filter such that 95% of bases were required to have a Phred score of 30 or higher (Available at http://hannonlab.cshl.edu/fastx_toolkit/index.html). SNPs against the *S. typhimurium* ST19 genome (available from the Prokaryotic Genome Analysis Tool http://tools.uwgenomics.org/pgat/), a derivative of *S. enterica* Typhimurium LT2 (GenBank: NC_003197.2) were then identified using BreSeq (Deatherage and Barrick, 2014). For Sanger sequencing, amplification of *rpoB* and *folA* loci was performed using Phusion DNA polymerase (ThermoFisher). PCR products were purified using the QIAquick PCR Purification Kit (QIAGEN) and sequencing was subsequently performed to identify mutations.

#### Bacterial 2-hybrid assays

Bacterial 2-hybrid assays were performed as previously described (Dove et al., 1997). Briefly, domains from the genes of interest were fused to the Lambda repressor (cI) and the N-terminal domain of *E. coli* RNA polymerase’s alpha subunit (α-NTD) using the plasmids pACλCI and pBRα, respectively. These fusion constructs were transformed into *E. coli* containing the Lambda operator sequence inserted upstream of a luciferase reporter (NanoLuc, Promega) using an F’ episome. For expression of fusion constructs, cells were grown overnight in LB + 20μM IPTG at 30°C and diluted 1:100 intro fresh LB + 20μM IPTG at 30°C the next morning and were grown until OD_600_ = 2. For relative light unit measurements, Nano-glo substrate (Promega) was added to cultures according to the manufacturer’s instructions and luminescence was measured in a SpectraMax M3 96-well plate reader.

#### DNA damage survival assays

For both *S. typhimurium* and *B. subtilis*, cultures were started from single colonies and harvested at exponential growth (OD_600_ = 0.3-0.6). To determine 4-Nitro-Quinolone Oxide (4-NQO) survival, cell dilutions were spotted onto LB agar plates (for CFU enumeration) and LB agar plates containing either 0.2 μM (*B. subtilis*) or 4 μM (*S. typhimurium*) 4-NQO. To determine UV sensitivity, cells were spotted onto LB agar plates and exposed to the indicated intensity of UV light using a Mineralight XX 15V UV light source (UVP). Surviving colonies were enumerated after overnight incubation at 30°C.

#### Bone Marrow-Derived Macrophage (BMM) infections

BMMs were derived from BALB/c mice as previously described (Weischenfeldt and Porse, 2008). All protocols for harvesting BMMs were reviewed and approved by the Institutional Animal Care and Use Committee at the University of Washington. BMMs were cultured in RPMI media with 10% heat-inactivated FBS at 37°C in 5% CO_2_. Approximately 10^6^ BMMs were plated overnight in 24-well plates at 37°C in 5% CO_2_. For infections, a single colony of *S. typhimurium* was picked and grown overnight at 37°C in LB, diluted back to an OD_600_ = 0.05 the following day and grown at 37°C in LB until cultures reached OD_600_ = 0.5. Bacteria were then washed 2x with 1X PBS and resuspended in RPMI +10% FBS and inoculated at 10:1 multiplicity of infection with BMMs at 37°C in 5% CO_2_ for 30 min. Plates were then washed 2x with 1X PBS and RPMI +10% FBS + 50 μg/mL gentamicin was added for killing of extracellular bacteria. Infected macrophages were lysed at indicated time points with 1X PBS + 0.1% Triton X-100 and plated on LB for CFU enumeration.

### Quantification and Statistical Analysis

The definition of all data points, variance measurement, and statistical tests used are included in each figure legends. The number of replicates for each experiment are also described in each figure legend. Statistical measurements were performed in Prism 7.0 (Graphpad).

### Data and Software Availability

The accession number for the data reported in this paper is NCBI SRA: PRJNA492467, SRA:SUB4542953.

## Supplemental Information

Document S1. Figures S1–S5

Table S1. Mutations Identified from Trimethoprim Evolution using WGS, Related to Figures 2, S2, and S3 Catalog of mutations for each replicate (6 WT and 6 Δmfd) at each time course of trimethoprim evolution (7 total). Highlighted in yellow are folA resistance conferring mutations (including putative promoter mutations), and highlighted in blue are dnaQ hypermutator mutations. For each mutation, the genomic location, nucleotide change, amino acid change, and gene are listed. Each individual replicate sequenced is cross-referenced with Figure S2, and isolates used to measure mutation rate in Figure S3 are indicated.

Table S2. Bacterial Strains, Strain Construction Details, and Plasmids Used in This Study, Related to STAR Methods Experimental Model and Subject Details Section

Table S3. Primers Used in This Study, Related to STAR Methods Experimental Model and Subject Details Section and Key Resources Table Oligonucleotides Section

Document S2. Article plus Supplemental Information
