# Supplementary material for: Inhibiting the Evolution of Antibiotic Resistance
Source: Mol Cell. 2019 Jan 3;73(1):157–165.e5. doi: 10.1016/j.molcel.2018.10.015 (PMC6320318; doi:10.1016/j.molcel.2018.10.015)
Supplement: Document S1. Figures S1–S5 [file mmc1.pdf]

Molecular Cell, Volume *73*

Supplemental Information

Inhibiting the Evolution of Antibiotic Resistance

Mark N. Ragheb, Maureen K. Thomason, Chris Hsu, Patrick Nugent, John Gage, Ariana N. Samadpour, Ankunda Kariisa, Christopher N. Merrikh, Samuel I. Miller, David R. Sherman, and Houra Merrikh

Figure S1

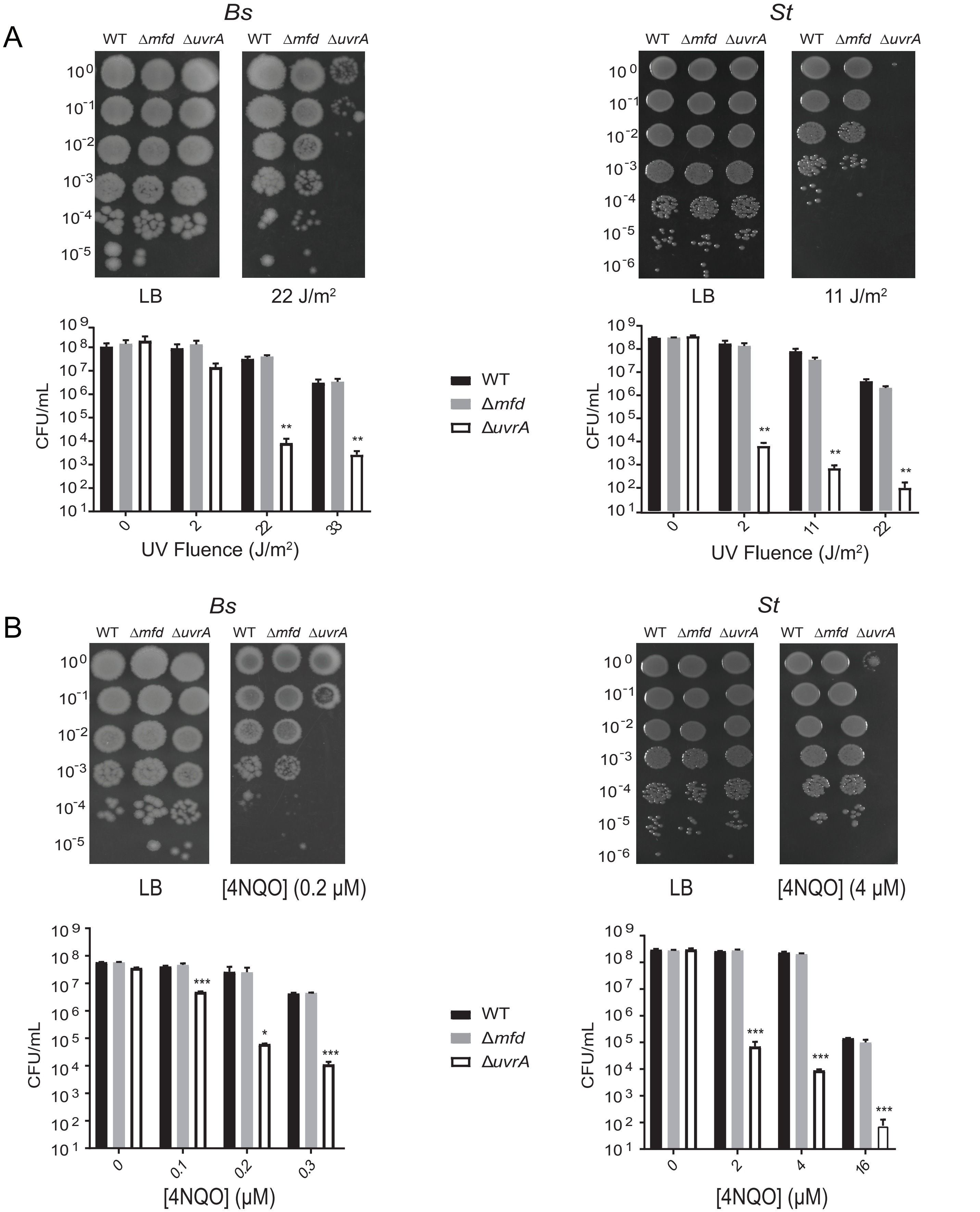

Figure S1. Cells lacking Mfd are not significantly sensitive to DNA damaging agents, Related to Figure 1
Survival assays to (A) UV damage and (B) 4NQO for WT, Δ*mfd*, and Δ*uvrA* strains of *B. subtilis* HM1 (Bs) and *S. typhimurium* ST19 (St). (*uvrA* knockouts, known to be sensitive to DNA damage, were included for comparison to *mfd* knockouts). Data represents at least two independent experiments with duplicates for each experiment. Errors bars indicate s.e.m. Statistical significance was determined using two-tailed Student's t-test (*p-value <0.05, **p-value <0.01, ***p-value <0.001).

Figure S2

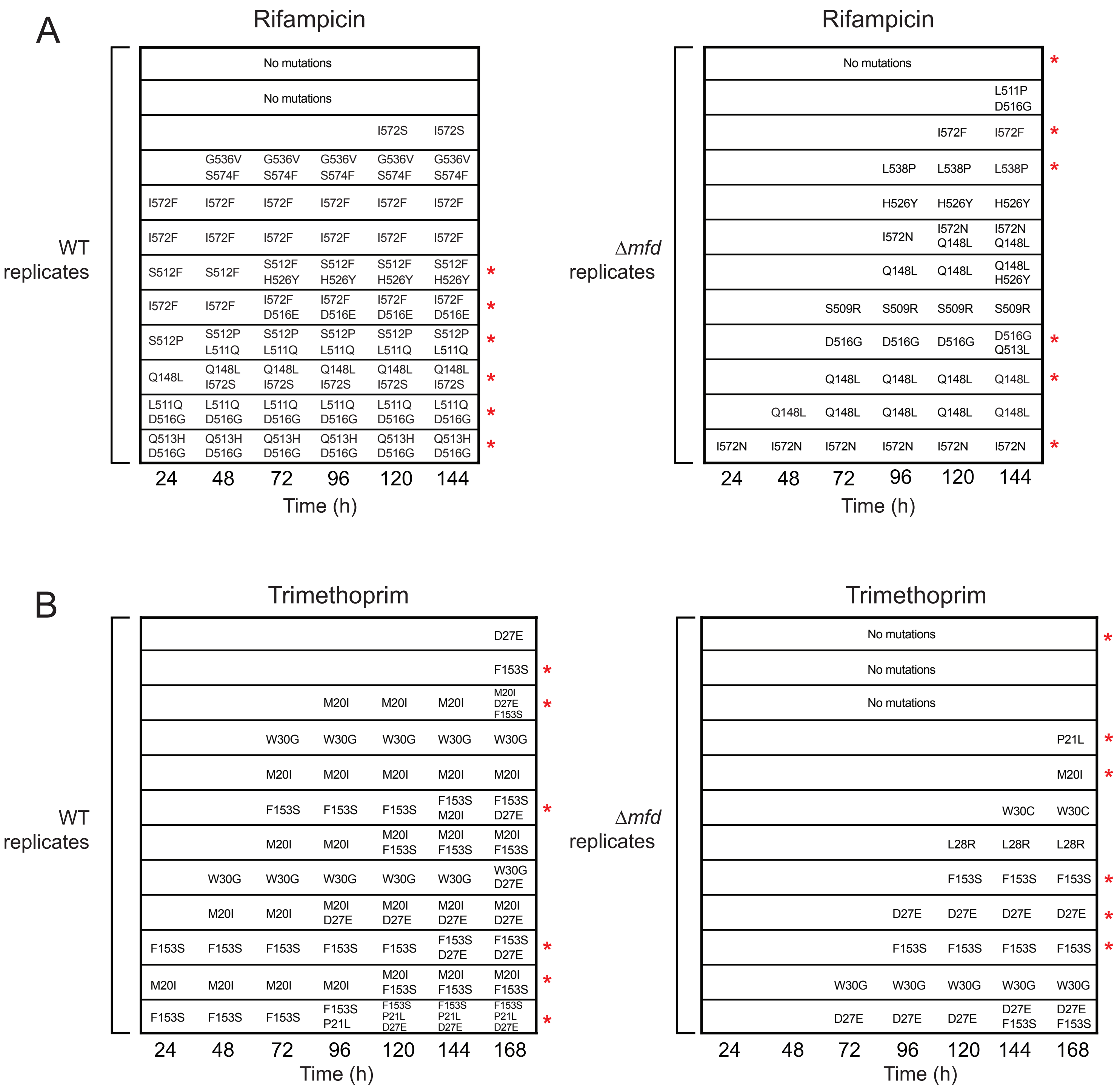

Figure S2. Cells lacking Mfd show fewer and delayed resistance-conferring mutations, Related to Figure 2, Figure S3, and Table S1

(A) Sequencing of *rpoB* was performed at each time point from rifampicin evolution assays to identify mutations that confer resistance in WT and Δ*mfd* strains of *S. typhimurium* (12 replicates per strain). (B) Sequencing of *folA* was performed at each time point from trimethoprim evolution assays to identify mutations that confer resistance in WT and Δ*mfd* strains of *S. typhimurium* (12 replicates per strain). Shown are the position and corresponding amino acid changes. *Indicates replicates sequenced by whole genome sequencing.

Figure S3

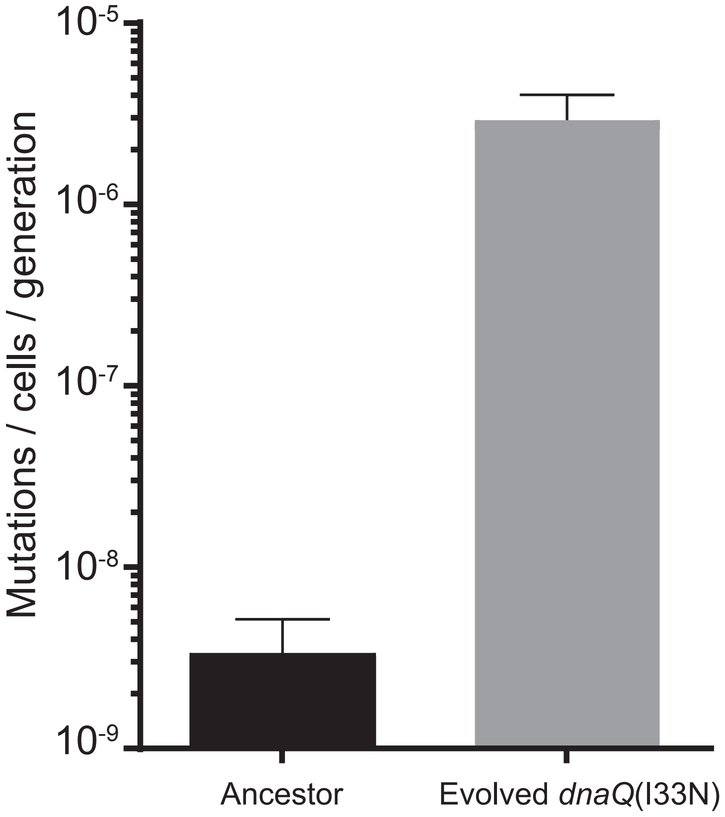

Figure S3. Development of hypermutation in evolved WT strains of *S. typhimurium*, Related to Figure 2, Figure S2, and Table S1
Mutation rate analysis of *S. typhimurium* strains evolved to trimethoprim. Assays were performed on rifampicin plates as described in Figure 1. The individual ancestor and evolved WT (containing a *dnaQ*133N mutation) isolates used in this experiment are indicated in Table S1. The number of replicates per isolate is 12.

Figure S4

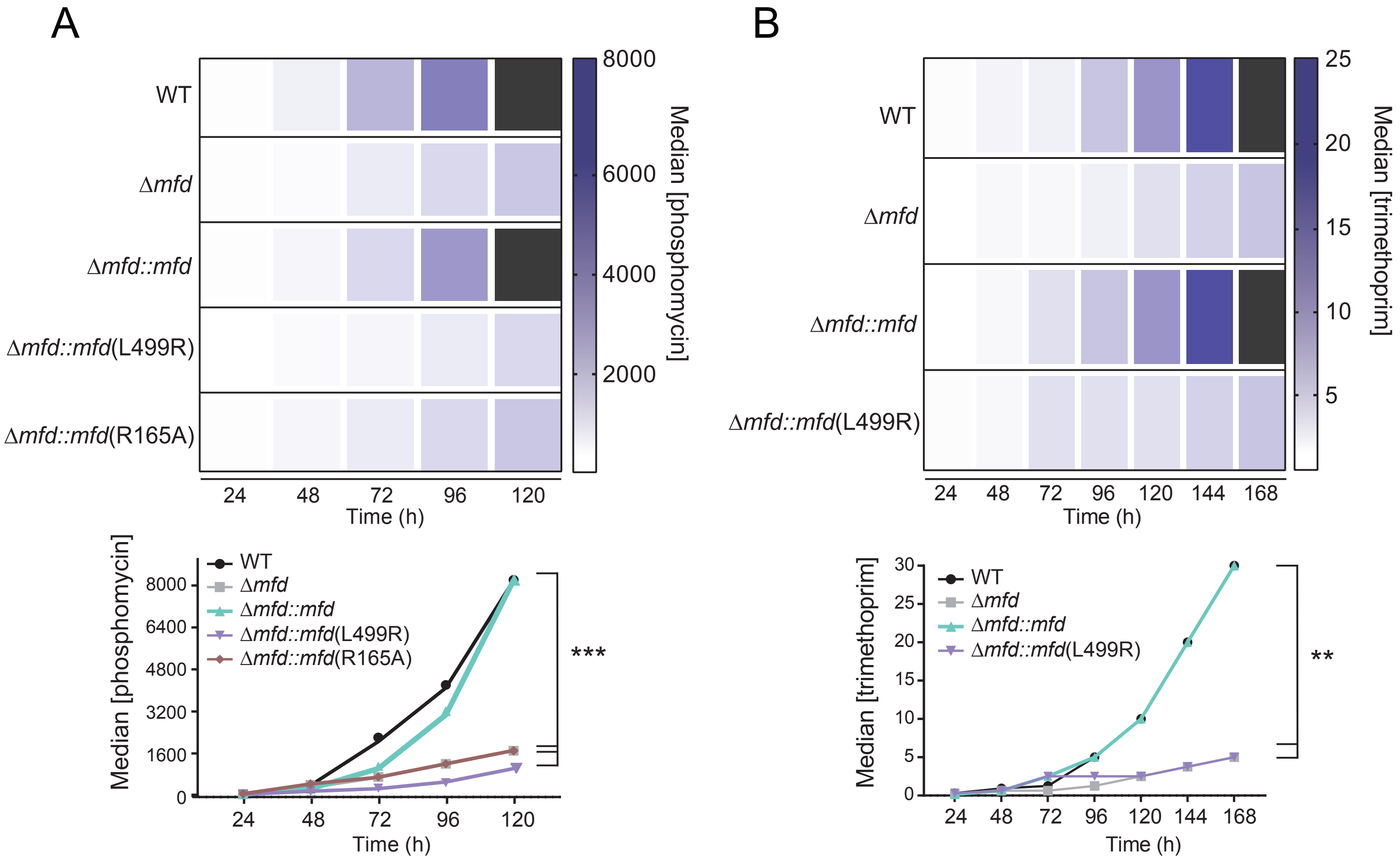

Figure S4. Mfd requires interaction with RNAP and UvrA to promote evolution to antibiotics, Related to Figure 4

Evolution of indicated *S. typhimurium* ST19 strains to phosphomycin (A) and trimethoprim (B). Plots and statistical testing for evolution assays were performed as described in Figure 2. ***p-value <0.001 between WT and Δ*mfd*, between WT and Δ*mfd::mfd*(L499R), and between WT and Δ*mfd::mfd*(R165A) strains for evolution to phosphomycin. **p-value <0.01 between WT and Δ*mfd* and between WT and Δ*mfd::mfd*(L499R) strains for evolution to trimethoprim. n = 12-24 replicates per strain.

Figure S5

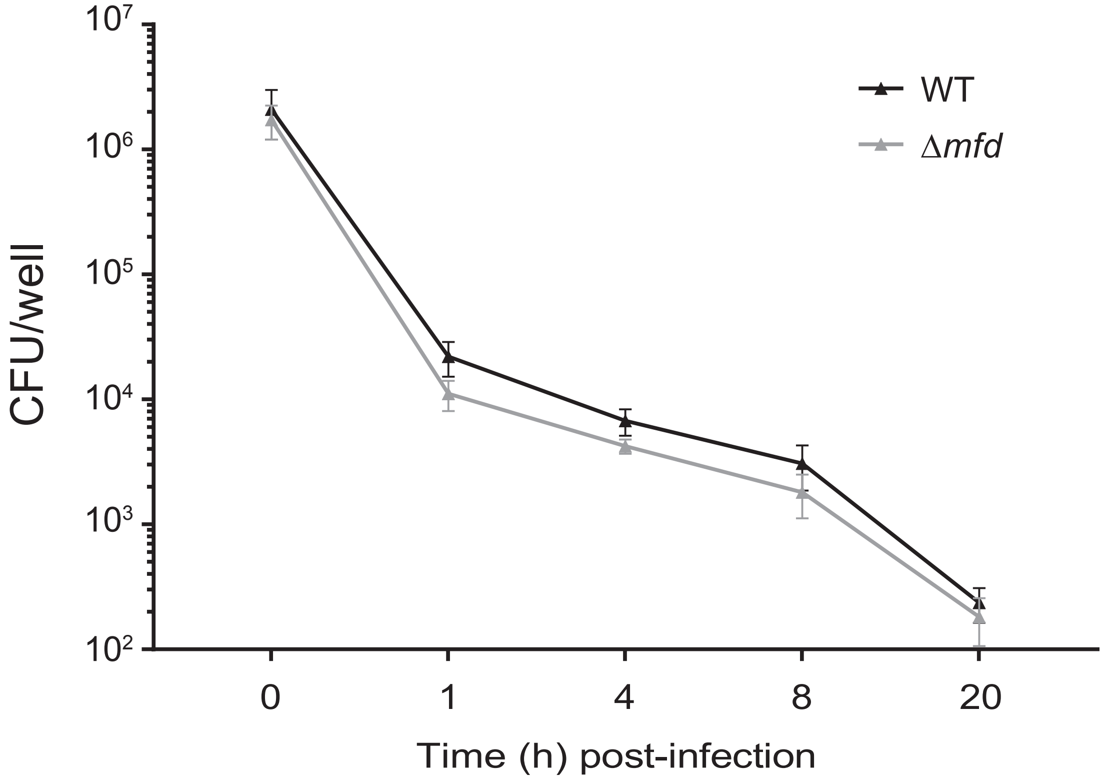

Figure S5. Strains lacking Mfd show no survival defects in bone marrow macrophages, Related to Figure 1

Murine-derived bone marrow macrophages (BMMs) were infected with WT and Δ*mfd* strains of *S. typhimurium* ST19 and harvested for CFU enumeration at indicated times points. Data represents two independent experiments with triplicate samples for each given experiment. Error bars indicate s.e.m.
